# Impact of mammographic screening and advanced cancer definition on the percentage of advanced-stage cancers in a steady-state breast screening programme in the Netherlands

**DOI:** 10.1038/s41416-020-0968-6

**Published:** 2020-07-09

**Authors:** Linda de Munck, Sabine Siesling, Jacques Fracheboud, Gerard J. den Heeten, Mireille J. M. Broeders, Geertruida H. de Bock

**Affiliations:** 1 Department of Research and Development, Netherlands Comprehensive Cancer Organisation, Utrecht, The Netherlands; 2 Department of Epidemiology, University of Groningen, University Medical Center Groningen, Groningen, The Netherlands; 3 Department of Health Technology & Services Research, Technical Medical Centre, University of Twente, Enschede, The Netherlands; 4 Department of Public Health, Erasmus MC, University Medical Center Rotterdam, Rotterdam, The Netherlands; 5 Dutch Expert Centre for Screening, Nijmegen, The Netherlands; 6 Department of Radiology, Amsterdam UMC, University of Amsterdam, Amsterdam, The Netherlands; 7 Radboud Institute for Health Sciences, Radboud University Medical Center, Nijmegen, The Netherlands

**Keywords:** Breast cancer, Population screening, Cancer screening, Epidemiology, Breast cancer

## Abstract

**Background:**

To estimate the percentages of advanced-stage breast cancers (BCs) detected during the course of a steady-state screening programme when using different definitions of advanced BC.

**Methods:**

Data of women aged 49–74 years, diagnosed with BC in 2006–2015, were selected from the Netherlands Cancer Registry and linked to the screening registry. BCs were classified as screen-detected, interval or non-screened. Three definitions of advanced BC were used for comparison: TNM stage (III–IV), NM stage (N+ and/or M+) and T size (invasive tumour ≥15 mm). Analyses were performed assuming a 10% overdiagnosis rate. In sensitivity analyses, this assumption varied from 0 to 30%.

**Results:**

We included 46,734 screen-detected, 17,362 interval and 24,189 non-screened BCs. By TNM stage, 4.9% of screen-detected BCs were advanced, compared with 19.4% and 22.8% of interval and non-screened BCs, respectively (*p* < 0.001). Applying the other definitions led to higher percentages of advanced BC being detected. Depending on the definition interval, non-screened BCs had a 2–5-times risk of being advanced.

**Conclusion:**

Irrespective of the definition, screen-detected BCs were less frequently in the advanced stage. These findings provide evidence of a stage shift to early detection and support the potential of mammographic screening to reduce treatment-related burdens and the mortality associated with BC.

## Background

Breast cancer (BC) is the most common cancer among women, and tumour stage at diagnosis is important to overall survival.^1–3^ Early diagnosis results in a mean lower tumour stage, which allows for better treatment options and ultimately reduces mortality. Although BC screening by mammography was introduced based on these arguments, there is ongoing debate as to whether screening affects the occurrence of advanced-stage BC.^4–6^

In a previous study, we studied the incidence rates of advanced stage, and found that there was a lower incidence of advanced BCs in screened women than in non-screened women, with estimates of 38 and 94 BCs per 100,000 women, respectively.^7^ Most other studies have assessed the rates of advanced BCs in the total target population and/or have compared the percentages of early and advanced BCs in screen-detected versus other cancers.^8–11^ However, these studies used different definitions for BC staging, making the true differences in percentage difficult to compare. Comparison has been further complicated by the potential for overdiagnosis. This is defined as the detection by screening of a BC (ductal carcinoma in situ or invasive carcinoma) that would never have presented clinically during a woman’s lifetime.^12^ A more favourable ratio between tumours of early and advanced stages can result not only from a reduction in the number of advanced BCs because of early detection and treatment, but also from an increase in overdiagnosis. At present, the extent of this overdiagnosis is unclear, with estimates ranging from 0 to 52%,^13–18^ and levels sitting at ~10% in the Netherlands.^13,18^

When comparing the published percentages of advanced BCs, reported differences in the impact of mammographic screening might, at least partly, be attributable to the varying definitions of advanced tumours. Therefore, we aimed to estimate the percentages of advanced BC in a steady-state biennial screening programme when using different definitions for advanced stage. We also wanted to assess the impact of different assumptions of overdiagnosis on the estimated percentages of advanced BC. These results will contribute to a clear perspective of what already has been published on this topic.

## Methods

### Study design

This population-based study included all women aged 49–74 years diagnosed with BC (invasive cancers and ductal carcinomas in situ) between January 1, 2006 and December 31, 2015. We used data from the Netherlands Cancer Registry (NCR), hosted by the Netherlands Comprehensive Cancer Organisation (IKNL),^1^ and linked them to data from the Netherlands Breast Cancer Screening Registry. Percentages were compared between screen-detected, interval and non-screened cases of BC, using three definitions of advanced BC (i.e., TNM, NM and T-size staging). The Central Committee on Research involving Human Subjects determined that this study did not require approval from an ethics committee. The study was approved by the Privacy Review Board of the NCR.

### Study population

We selected women aged 49–74 years who were diagnosed with BC (invasive and ductal carcinoma in situ) between January 1, 2006 and December 31, 2015 from the NCR. Their data were linked to those in the Netherlands Breast Cancer Screening Program. The linkage data identified women screened between January 1, 2004 and December 31, 2015, to cover a period of at least 24 months before BC diagnosis. Given that the screening programme invited women for biennial screening, the 24-month threshold before diagnosis was considered important when defining the detection mode (i.e., the relation between BC diagnosis and the screening programme). For example, a woman diagnosed with BC in 2006 could have been screened in 2004 or 2005 and categorised with interval cancer, whereas if she had not attended the screening programme, the cancer would be categorised as a non-screened BC.

We excluded women diagnosed after their prevalent screen on the basis that screening is not a once-only event. Furthermore, we excluded women with lobular carcinoma in situ because this is not considered malignant. Women diagnosed with BC in 5 years before the current diagnosis were also excluded to minimise interference from hospital follow-up visits. For women with synchronous BC, only the most advanced cancer was included.

### Definitions of BC groups and cases

We defined three groups of BC: screen-detected, interval and non-screened. Screen-detected BCs included cases diagnosed within 24 months after being recalled for further diagnostic workup due to a positive screening result; interval BCs included cases diagnosed within 24 months after a negative screening result, which indicates no recall necessary. Non-screened BCs included those diagnosed in women at a screening interval beyond the planned 24 months (i.e., not recently screened) or never attended screening, as we were not able to divide these two groups.

Cases of advanced BC were identified using three definitions: TNM staging, NM staging and T-size staging. Using the TNM classification, stages III–IV were defined as advanced cancer, and stages 0–II were defined as early cancer.^19,20^ Based on NM staging, tumours with positive lymph nodes and/or metastasis (N+ and/or M+) were defined as advanced NM stage, and tumours without positive lymph nodes or metastasis (N0M0) were defined as early NM stage.^21^ When using the tumour size only, an advanced T-size stage was defined as the presence of an invasive tumour measuring ≥15 mm, whereas an early T-size stage was defined as either a tumour measuring <15 mm or as a ductal carcinoma in situ (regardless of size).^22^ For each definition, BCs of unknown stage are included in the description of the cohort characteristics, but not in the statistical analyses. Furthermore, in sensitivity analyses, additional definitions of advanced TNM stage were included, in which advanced TNM stage was defined as stage IIB–IV (compared with 0–IIA) or as stage II–IV (compared with stage 0–I).

### Data sources

Data were accessed from the NCR and the Netherlands Breast Cancer Screening Program. In the Netherlands, all new cancer cases are registered in the NCR, which contains data on patient, tumour and treatment characteristics for all in situ and invasive malignancies diagnosed since 1989. The main source of notification for the NCR is the Nationwide Histopathology and Cytopathology Data Network and Archive (PALGA).^23^ After the NCR has been notified, specially trained registration clerks visit hospitals to collect information on patient and tumour characteristics, including the stage and treatment data, directly from patient records. Tumour topography, morphology and grade were coded according to the International Classification of Diseases for Oncology, 3rd edition.^24^ Staging is classified according to the TNM Classification of Malignant Tumours, using the sixth edition until 2009 and the seventh edition thereafter.^19,20^

The population-based Netherlands Breast Cancer Screening Program has been operational since 1990, initially inviting women aged 49–69 years for a biennial screening examination, but including women aged 70–74 years from 1998 onwards.^25^ All mammographic examinations are performed by specialist radiographers and are double-read by accredited radiologists. Recall for further diagnostic workup is indicated if the screening examination is incomplete (i.e., Breast imaging reporting and data system [BI-RADS] 0) or if there are suspicious or malignant findings (i.e., BI-RADS 4 or 5).^26,27^ Between 2003 and 2010, screen-film mammography has gradually been replaced by full-field digital mammography.^28^ Permission for linkage to the NCR was requested from women when they attended screening. This was based on an opt-out option, which was used by 0.02% of all women screened.^29^

### The impact of overdiagnosis

To consider the impact of possible overdiagnosis in our main analysis, we assumed an overdiagnosis estimate of 10%, consistent with that reported in the Netherlands.^13,18^ By definition, overdiagnosis only occurs in BCs that are detected by screening in an early stage. For all three definitions of advanced BC, we performed separate calculations to correct for overdiagnosis and performed separate analyses. For all screen-detected BCs, we assumed that 10% of the total sample was overdiagnosed, and then excluded this number at random from the early screen-detected BCs. However, given that the true overdiagnosis rate is unknown, and that published estimates differ substantially, we performed sensitivity analyses in which the assumed overdiagnosis estimates were 0 and 30%.^30^ The adjustments for overdiagnosis resulted in a lower total number of screen-detected BCs and in a higher percentage of advanced screen-detected BCs. To check whether exclusion was performed at random, the baseline characteristics of the remaining screen-detected BCs were compared with the original sample (Supplementary Table 1).

### Statistical methods

The percentage of advanced cancers in the screen-detected, interval and non-screened BC groups was compared by the Chi-squared test. Univariable and multivariable logistic regression analyses were used to estimate differences in the percentage of advanced disease among the three subgroups, controlling for age at diagnosis, year of diagnosis and socioeconomic status (SES). Data for the multivariable analyses are reported as odds ratios (ORs) with their 95% confidence intervals (95% CIs). Age at the time of diagnosis was categorised as 49–59, 60–69 and 70–74 years, with the age category 60–69 years defined as the reference group. SES was determined by education, household income and labour market status, based on postal codes, and categorised as high (reference) medium and low SES.^31^ Statistical significance was set at a *P* value of <0.05, and all tests were two-sided. Analyses were performed using the STATA Software Package, Version 14.1 for Windows (Stata Corporation LP, College Station, TX, USA).

## Results

### Participants

**Fig. 1:**
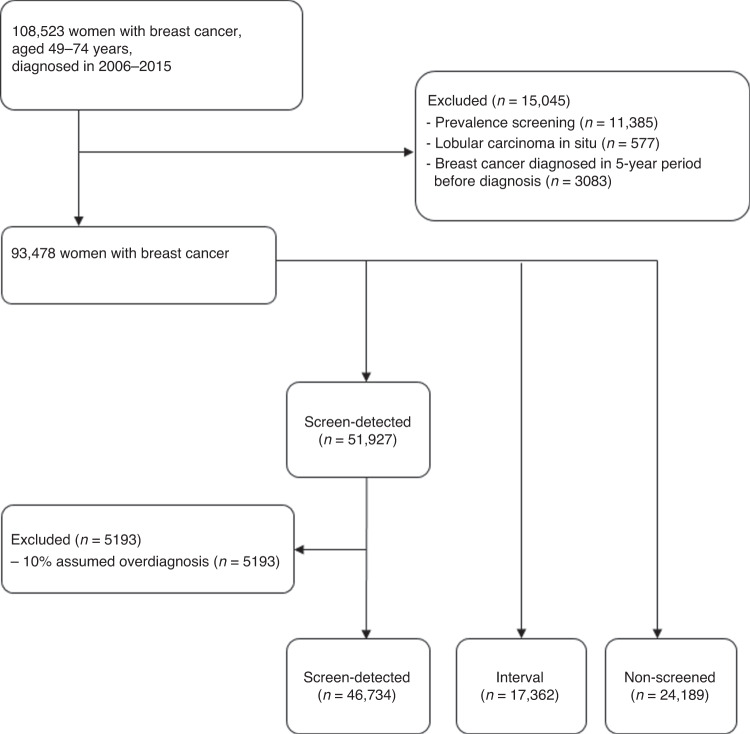
Flow chart of included patients. We included 88,285 BC cases, of which 46,734 were screen-detected, 17,362 were interval and 24,189 were non-screened. We excluded 5193 cases from the 51,927 initial cases of screen-detected BCs based on the assumption of a 10% overdiagnosis rate.

In our main analysis, we included 88,285 BC cases, of which 46,734 were screen-detected, 17,362 were interval and 24,189 were non-screened (Fig. 1). Note that among the 51,927 initial cases of screen-detected BCs, we excluded 5193 cases based on the assumption of a 10% overdiagnosis rate, leaving 46,734 screen-detected cases. Median time between a negative screening result and interval cancer diagnosis was 14 months (interquartile range 10 months). The baseline characteristics are shown in Table 1.

**Table 1:** Breast cancer characteristics for each detection cohort.

	Screen-detected		Interval		Non-screened		Total	*p* value*
	*N*	%	*N*	%	*N*	%	*N*	
*Age*								
Mean (IQR)	63 (58–68)		61 (56–67)		60 (53–67)			
49–59	15,610	33.4	6989	40.3	11,211	46.3	33,810	<0.001
60–69	22,192	47.5	7795	44.9	9107	37.6	39,094	
70–74	8932	19.1	2578	14.8	3871	16.0	15,381	
*Year*								
2006	3631	7.8	1598	9.2	2156	8.9	7385	<0.001
2007	3976	8.5	1618	9.3	2261	9.3	7855	
2008	4051	8.7	1661	9.6	2393	9.9	8105	
2009	4127	8.8	1698	9.8	2467	10.2	8292	
2010	4526	9.7	1756	10.1	2301	9.5	8583	
2011	4850	10.4	1832	10.6	2354	9.7	9036	
2012	5241	11.2	1825	10.5	2415	10.0	9481	
2013	5490	11.7	1737	10.0	2513	10.4	9740	
2014	5349	11.4	1865	10.7	2691	11.1	9905	
2015	5493	11.8	1772	10.2	2638	10.9	9903	
*SES*								
High (8–9–10)	14,486	31.0	5562	32.0	7623	31.5	27,671	<0.001
Medium (4–5–6–7)	18,681	40.0	7014	40.4	9472	39.2	35,167	
Low (1–2–3)	13,567	29.0	4786	27.6	7094	29.3	25,447	
Total	46,734	100	17,362	100	24,189	100	88,285	

*IQR* interquartile range, *SES* socioeconomic status.

*Chi-squared test.

### Advanced TNM stage (stages III and IV)

**Fig. 2:**
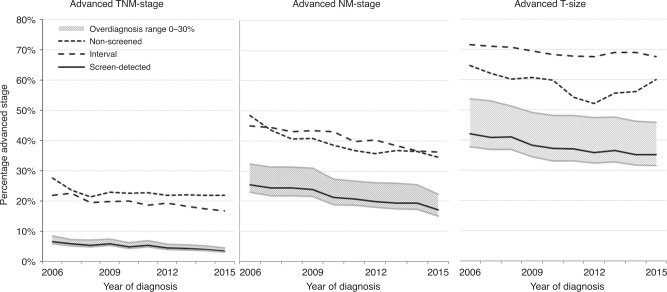
Percentages of advanced breast cancers over time in the screen-detected, interval and non-screened cohorts by three definitions of advanced stage. The solid line indicates the screen-detected cancers assuming 10% overdiagnosis. The shaded area then indicates the percentage assuming 0% overdiagnosis (lower limit) to 30% overdiagnosis (upper limit).

**Fig. 3:**
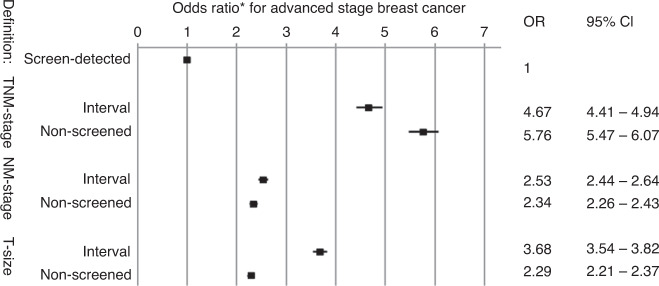
Odds ratios for advanced breast cancer between different cohorts by the three definitions of advanced stage. Data are for the interval and non-screened cohorts compared with the screen-detected cohort. *Multivariable analyses corrected for age, year of diagnosis and socioeconomic status. 95% CI, 95% confidence interval.

Based on TNM staging, 4.9% of screen-detected BCs were advanced (Table 2, Fig. 2). More cancers were diagnosed as being advanced stage in the interval (19.4%) and non-screened (22.8%) cohorts (*P* < 0.001). Multivariable logistic regression indicated that compared with screen-detected BC, there was an increased risk of the interval and non-screened BCs being advanced, with ORs of 4.67 (95% CI, 4.41–4.94) and 5.76 (95% CI, 5.47–6.07), respectively (Fig. 3). In sensitivity analyses using additional definitions of advanced TNM stage, the results remained similar (Supplementary Figs. 1 and 2).

**Table 2:** Percentages of early and advanced disease in each detection cohort, reported by definition of advanced-stage breast cancer.

	Screen-detected		Interval		Non-screened		Total	*p* value*
	*N*	%	*N*	%	*N*	%	*N*	
*TNM stage*								
Early stage (St 0–I–II)	44,368	95.1	13,953	80.6	18,578	77.2	76,899	<0.001
Advanced stage (St III–IV)	2272	4.9	3353	19.4	5491	22.8	11,116	
Unknown	94		56		120		270	
*NM stage*								
Early stage (N0M0)	35,391	78.8	9012	59.0	12,886	60.9	57,289	<0.001
Advanced stage (N+ and/or M+)	9514	21.2	6270	41.0	8285	39.1	24,069	
Unknown	1829		2080		3018		6927	
*T stage*								
Early stage (<15 mm)	28,467	62.4	4976	30.9	8858	41.8	42,301	<0.001
Advanced stage (invasive ≥15 mm)	17,133	37.6	11,107	69.1	12,353	58.2	40,593	
Unknown	1134		1279		2978		5391	
Total	46,734		17,362		24,189		88,285	

*Chi-squared test.

### Advanced NM stage (N+ and/or M+)

Based on NM staging, 21.2% of BCs were advanced in the screen-detected cohort, compared with 41.0% and 39.1% in the interval and non-screened cohorts, respectively (*P* < 0.001, Table 2). Analysis confirmed that interval and non-screened BCs were more often advanced, with the respective ORs of 2.53 (95% CI, 2.44–2.64) and 2.34 (95% CI, 2.26–2.43) (Fig. 2). Compared with TNM staging, the percentage of advanced cancers based on NM staging was higher for all detection modes, and the ORs for advanced BC were almost halved in the interval and non-screened cohorts (Figs. 2 and 3).

### Advanced T size (invasive tumours ≥15 mm)

When defining advanced BCs as invasive disease measuring ≥15 mm (i.e., by T size), 37.6% of BCs in the screen-detected cohort were considered advanced (Table 2, *P* < 0.001). The percentages of advanced BCs in the interval (69.1%) and non-screened (58.2%) cohorts were also significantly higher (*P* < 0.001). Compared with the screen-detected cohorts, the ORs for advanced BC based on T size were 3.68 (95% CI, 3.54–3.82) for the interval cohort and 2.29 (95% CI, 2.21–2.37) for the non-screened cohort (Fig. 3). Compared with TNM staging, the percentage of advanced BC identified by T-size staging was higher for all detection modes, and the ORs for advanced T-size BC were lower in the interval and non-screened cohort.

### The impact of different rates of cancer overdiagnosis

The effect of overdiagnosis was further explored by assuming either no overdiagnosis (0%) or higher overdiagnosis estimates (30%). However, regardless of the estimate used, the percentages of advanced cancers remained significantly higher in the interval and non-screened BC cohorts (Fig. 2).

When assuming no overdiagnosis, the percentages of advanced disease decreased in all instances: for TNM staging, it changed from 4.9 to 4.4% (Fig. 2, Supplementary Table 2), for NM staging, it changed from 21.2 to 19.0% and for T-size staging, it changed from 37.6 to 33.7%. Compared with screen-detected BC, the ORs for advanced stage were also significantly higher for interval and non-screened BCs when using all definitions (Supplementary Fig. 3). Overdiagnosis only affected the percentage of screen-detected BC, and the percentages of advanced-stage BC remained similar in the interval and non-screened cohorts.

In contrast to the 0% estimate, defining 30% of all screen-detected BCs as overdiagnosed resulted in an increase in the percentages of advanced screen-detected BC compared with the 10% assumption. However, for all three definitions of advanced stage, the percentage of advanced BCs in the screen-detected cohort remained significantly lower than in the interval and non-screened cohorts. The ORs for advance stage remained significantly higher for interval and non-screened BCs when using all definitions (Supplementary Fig. 4).

## Discussion

Screen-detected cancers were less often diagnosed at an advanced stage compared with interval and non-screened cancers, regardless of the definition used for advanced BC. Indeed, compared with the screen-detected cohort, the interval and non-screened cohorts were 2–3 times and 2–5 times more likely to be advanced BC, respectively. When exploring the impact of overdiagnosis, even with the assumption of a 30% overdiagnosis estimate, there remained significantly higher percentages of advanced BCs in the interval and non-screened cohorts.

Several studies have been performed to identify the percentages or incidence rates of early and advanced BC identified by screening, based on individual data.^5,8,9,11,14,32–34^ Using different definitions of advanced stage, each of these studies has shown lower percentages of advanced BC in screen-detected cohorts. Furthermore, studies that have used TNM staging^11,33,34^ have showed larger differences in advanced BC than those that have used NM staging,^8,9,14^ which is consistent with our results. One study of advanced BC reported comparable differences when using definitions comparable to those in the current research.^32^ Comparing ever-screened women to non-screened women aged 50–64 years, they reported an OR of 0.41 when defining advanced BC as stage IIB or higher, and 0.67 when using the NM-stage definition. The current study shows a direct comparison of the effect of breast screening on the percentage of advanced stage for each definition. Our results indicate that the larger differences found in other studies using TNM stage are indeed partly attributable to the definition used.

Overdiagnosis results in an artificial decrease in the percentage of advanced cancers. Such a decrease can result from an actual reduction in the number of advanced tumour stages or from an increase in the number of early tumour stages, which itself may be due to overdiagnosis. Most likely, both factors contributed to the reduction in advanced BC seen in our study. In previous research, we showed that there was a substantially lower incidence rate of advanced cancers among screened compared with non-screened women, and this difference in incidence rate was unaffected by overdiagnosis.^7^ However, most studies that published percentages to date have concluded that overdiagnosis does play a role. In the present study, we assumed a 10% rate of overdiagnosis among screen-detected cancers to study the percentage of women with advanced BC accurately in the Dutch population.

A potential limitation of this study is that we had no information about women with a higher-than- average risk for BC (e.g., those with *BRCA1/2* mutations or a high familial risk), so we cannot confirm if these women attended screening. However, because women younger than 49 years were not included, we doubt that this will have affected our conclusions. A second limitation is that participation in the screening programme is voluntary, meaning that certain factors may have influenced attendance. Although women with a low SES have lower attendance rates,^35^ we identified that this subgroup was more likely to be diagnosed with advanced cancer and corrected for SES in the multivariable analysis. In addition, the overall influence of bias due to self-selection, on the effectiveness of the Dutch screening programme, has been shown to be minor.^36^ Finally, we obtained no structural information about breast density, which is known to reduce mammographic detectability, and to increase the risk of BC.^37,38^ Unfortunately, the extent of this effect on our results is unknown.

The major strengths of this study are the population-based design covering the entire country, and the fact that we were able to link data from the cancer registry to those from the screening registry at the individual level. The same study population was also used for all definitions, enabling a direct comparison of the effect of breast screening on the percentage of advanced stage for each definition. Furthermore, BC cohorts were classified as screen-detected, interval and non-screened based on actual data for screening attendance. To minimise interference with hospital checkups, we only included first and second cancers diagnosed at least 5 years after the first BC, we only studied the effects of BC stage in a steady-state situation and we excluded women diagnosed after their prevalent screen. Although the exact magnitude of overdiagnosis cannot be known for certain, we were able to show a consistent effect of screening on advanced BC for a wide range of overdiagnosis rates.

## Conclusion

Irrespective of the definition used for advanced BC, screen-detected cohorts show lower rates of advanced BC than interval and non-screened cohorts. Our results support the hypothesis that mammographic screening causes a stage shift towards the diagnosis of early breast cancer stages, giving it the potential to reduce BC-related mortality and treatment-related burdens.

## Supplementary information

Supplemental material

## Data Availability

The data used in this study are available with permission of the Netherlands Cancer Registry.

## References

[1] IKNL Netherlands Cancer Registry data and figures. Utrecht, IKNL, https://www.iknl.nl/nkr-cijfers (2019).

[2] Saadatmand S, Bretveld R, Siesling S, Tilanus-Linthorst MMA (2015). Influence of tumour stage at breast cancer detection on survival in modern times: population based study in 173 797 patients. BMJ.

[3] Sopik V, Nofech-Mozes S, Sun P, Narod SA (2016). The relationship between local recurrence and death in early-stage breast cancer. Breast Cancer Res. Treat..

[4] Autier P, Boniol M, Middleton R, Doré JF, Héry C, Zheng T (2011). Advanced breast cancer incidence following population-based mammographic screening. Ann. Oncol..

[5] Broeders MJM, Allgood P, Duffy SW, Hofvind S, Nagtegaal ID, Paci E (2018). The impact of mammography screening programmes on incidence of advanced breast cancer in Europe: a literature review. BMC Cancer.

[6] de Glas NA, de Craen AJM, Bastiaannet E, Op’t Land EG, Kiderlen M, van de Water W (2014). Effect of implementation of the mass breast cancer screening programme in older women in the Netherlands: population based study. Br. Med. J..

[7] de Munck L, Fracheboud J, de Bock GH, den Heeten GJ, Siesling S, Broeders MJM (2018). Is the incidence of advanced‐stage breast cancer affected by whether women attend a steady‐state screening program?. Int. J. Cancer.

[8] Shen Y, Yang Y, Inoue LYT, Munsell MF, Miller AB, Berry DA (2005). Role of detection method in predicting breast cancer survival: analysis of randomized screening trials. J. Natl. Cancer Inst..

[9] Morrell S, Taylor R, Roder D, Robson B, Gregory M, Craig K (2017). Mammography service screening and breast cancer mortality in New Zealand: a National Cohort Study 1999–2011. Br. J. Cancer.

[10] Pálka I, Kelemen G, Ormándi K, Lázár G, Nyári T, Thurzó L (2008). Tumor characteristics in screen-detected and symptomatic breast cancers. Pathol. Oncol. Res.

[11] Fracheboud J, Otto SJ, van Dijck JAAM, Broeders MJM, Verbeek ALM, de Koning HJ (2004). Decreased rates of advanced breast cancer due to mammography screening in The Netherlands. Br. J. Cancer.

[12] International Agency for Research on Cancer. *Breast Cancer Screening*, 2nd edn. (International Agency for Research on Cancer, Lyon, 2016).

[13] de Gelder R, Heijnsdijk EAM, van Ravesteyn NT, Fracheboud J, Draisma G, de Koning HJ (2011). Interpreting overdiagnosis estimates in population-based mammography screening. Epidemiol. Rev..

[14] Bleyer A, Welch HG (2012). Effect of three decades of screening mammography on breast-cancer incidence. N. Engl. J. Med.

[15] Martinez-Alonso M, Vilaprinyo E, Marcos-Gragera R, Rue M (2010). Breast cancer incidence and overdiagnosis in Catalonia (Spain). Breast Cancer Res..

[16] Ripping TM, Verbeek ALM, Frachboud J, de Koning HJ, van Ravesteyn NT, Broeders MJM (2015). Overdiagnosis by mammographic screening for breast cancer studied in birth cohorts in The Netherlands. Int. J. Cancer.

[17] Nelson HD, Pappas M, Cantor A, Griffin J, Daeges M, Humphrey L (2016). Harms of breast cancer screening: Systematic review to update the 2009 u.s. preventive services task force recommendation. Ann. Intern. Med..

[18] Puliti Donella D (2012). Overdiagnosis in mammographic screening for breast cancer in Europe: a literature review. J. Med. Screen..

[19] Sobin, L. H. & Wittekind, C. (eds). T*NM Classification of Malignant Tumours*. 6th edn. (Wiley-Liss, New York, 2002).

[20] Sobin, L. H., Gospodarowicz, M. K. & Wittekind, C. (eds). *TNM Classification of Malignant Tumours, Seventh edition*. (Wiley-Blackwell, New York, 2009).

[21] Ruhl, J. L., Callaghan, C., Hurlbut, A., Ries, L. A. G., Adamo, P., Dickie, L. & Schussler, N. (eds). *Summary Stage 2018: Codes and Coding Instructions*. (National Cancer Institute, Bethesda, MD, 2018).

[22] Tabar L, Fagerberg G, Day NE, Duffy SW, Kitchin RM (1992). Breast cancer treatment and natural history: new insights from results of screening. Lancet.

[23] Casparie M, Tiebosch ATMG, Burger G, Blauwgeers H, van de Pol A, van Krieken JHJM (2007). Pathology databanking and biobanking in The Netherlands, a central role for PALGA, the nationwide histopathology and cytopathology data network and archive. Cell. Oncol..

[24] Fritz A (2000). International Classification of Diseases for Oncology.

[25] Fracheboud J, de Koning HJ, Boer R, Groenewoud JH, Verbeek AL, Broeders MJM (2001). Nationwide breast cancer screening programme fully implemented in The Netherlands. Breast.

[26] D’Orsi, C. J., Bassett, L. W., Berg, W., Feig, S. A., Jackson, V. P., Kopans, D. B., et al. (eds). *Breast Imaging Reporting and Data System: ACR BI-RADS Mammography*. 4th edn (American College of Radiology, Reston, VA, 2003).

[27] Timmers JMH, van Doorne-Nagtegaal HJ, Zonderland HM, van Tinteren H, Visser O, Verbeek ALM (2012). The breast imaging reporting and data system (BI-RADS) in the Dutch breast cancer screening programme: its role as an assessment and stratification tool. Eur. Radiol..

[28] van Luijt PA, Fracheboud J, Heijnsdijk EAM, den Heeten G, de Koning HJ (2013). Nation-wide data on screening performance during the transition to digital mammography: Observations in 6 million screens. Eur. J. Cancer.

[29] Fracheboud J, van Luijt PA, Sankatsing VDV, Ripping TM, Broeders MJM, Otten JDM (2014). National Evaluation of Breast Cancer Screening in the Netherlands 1990-2011/2012..

[30] Independent UK (2012). Panel on Breast Cancer Screening. The benefits and harms of breast cancer screening: an independent review. Lancet.

[31] van Duin C, Keij I (2002). Sociaal-economische status indicator op postcodeniveau [in Dutch]. Maandstat. van. de. Bevolk..

[32] Norman S, Localio AR, Zhou L, Weber A, Coates R, Malone K (2006). Benefit of screening mammography in reducing the rate of late-stage breast cancer diagnoses (United States).. Cancer Causes Control.

[33] Oberaigner W, Geiger-Gritsch S, Edlinger M, Daniaux M, Knapp R, Hubalek M (2017). Reduction in advanced breast cancer after introduction of a mammography screening program in Tyrol/Austria. Breast.

[34] Solin LJ, Schultz DJ, Kessler HB, Hanchak NA (1999). Downstaging of breast carcinomas in older women associated with mammographic screening. Breast J..

[35] Aarts MJ, Voogd AC, Duijm LEM, Coebergh JWW, Louwman WJ (2011). Socioeconomic inequalities in attending the mass screening for breast cancer in the south of the Netherlands—associations with stage at diagnosis and survival. Breast Cancer Res. Treat..

[36] Paap E, Verbeek AL, Puliti D, Broeders MJM, Paci E (2011). Minor influence of self-selection bias on the effectiveness of breast cancer screening in case-control studies in the Netherlands. J. Med. Screen.

[37] Boyd NF, Guo H, Martin LJ, Sun L, Stone J, Fishell E (2007). Mammographic density and the risk and detection of breast cancer. N. Engl. J. Med..

[38] van der Waal D, Emaus MJ, Bakker MF, den Heeten GJ, Karssemeijer N, Pijnappel RM (2015). Geographic variation in volumetric breast density between screening regions in the Netherlands. Eur. Radiol..

