# Supplementary material for: Impact of mammographic screening and advanced cancer definition on the percentage of advanced-stage cancers in a steady-state breast screening programme in the Netherlands
Source: Br J Cancer. 2020 Jul 9;123(7):1191–7. doi: 10.1038/s41416-020-0968-6 (PMC7524754; doi:10.1038/s41416-020-0968-6)
Supplement: Supplementary file 1 — Supplemental material [file 41416_2020_968_MOESM1_ESM.pdf]

Supplementary Table 1. Breast cancer characteristics within the screen-detected cancers after excluding the percentage of assumed overdiagnosis.

	Screen-detected cancers 10% overdiagnosis	Screen-detected cancers 0% overdiagnosis*	30% overdiagnosis
	N (%)	N (%)	N (%)
**Age**			
- mean (IQR)	63 (58-68)	63 (58-68)	63 (57-68)
49-59	15,610 (33.4)	17,351 (33.4)	12,294 (33.8)
60-69	22,194 (47.5)	24,695 (47.6)	17,176 (47.3)
70-74	8,932 (19.1)	9,881 (19.0)	6,879 (18.9)
**Year**			
2006	3,631 (7.8)	4,036 (7.8)	2,820 (7.8)
2007	3,976 (8.5)	4,444 (8.6)	3,151 (8.7)
2008	4,051 (8.7)	4,467 (8.6)	3,140 (8.6)
2009	4,127 (8.8)	4,577 (8.8)	3,282 (9.0)
2010	4,526 (9.7)	4,999 (9.6)	3,487 (9.6)
2011	4,850 (10.4)	5,369 (10.3)	3,707 10.3)
2012	5,241 (11.2)	5,807 (11.2)	4,083 (11.2)
2013	5,490 (11.7)	6,141 (11.8)	4,262 (11.7)
2014	5,349 (11.4)	5,967 (11.5)	4,169 (11.5)
2015	5,493 (11.8)	6,120 (11.8)	4,248 (11.7)
**SES**			
High (8-9-10)	14,486 (31.0)	16,105 (31.0)	11,268 (31.0)
Medium (4-5-6-7)	18,681 (40.0)	20,800 (40.1)	14,521 (33.9)
Low (1-2-3)	13,567 (29.0)	15,022 (28.9)	10,560 (29.1)
Total	46,734 (100)	51,927 (100)	36,349 (100)

Abbreviations: IQR: interquartile range; SES: socioeconomic status
* 0% overdiagnosis means that all patients are included in the analysis

Supplementary Table 2.
Percentage early- and advanced-stage disease of the remaining selection of screen-detected cancers after excluding the percentage of assumed overdiagnosis.

	Screen-detected cancers 10% overdiagnosis	Screen-detected cancers 0% overdiagnosis*	30% overdiagnosis
	N (%)	N (%)	N (%)
**TNM-Stage**			
Early-stage (St 0-I-II)	44,368 (95.1)	49,561 (95.6)	33,983 (93.7)
Advanced-stage (St III- IV)	2,272 (4.9)	2,272 (4.4)	2,272 (6.3)
Unknown	94	94	94
**NM-Stage**			
Early-stage (N0M0)	35,391 (78.8)	40,584 (81.0)	25,006 (72.4)
Advanced-stage (N+ and/or M+)	9,514 (21.2)	9,514 (19.0)	9,514 (27.6)
Unknown	1,829	1,829	1,829
**T-size**			
Early-stage (<15mm)	28,467 (62.4)	33,660 (66.3)	18,082 (51.3)
Advanced-stage (≥15mm)	17,133 (37.6)	17,133 (33.7)	17,133 (48.7)
Unknown	1,134	1,134	1,134
Total	46,734 (100)	51,927 (100)	36,349 (100)

* 0% overdiagnosis means that all patients are included

Supplementary Figure 1. Percentages of advanced breast cancers over time for two other definitions of advanced TNM-stage, compared to the main definition

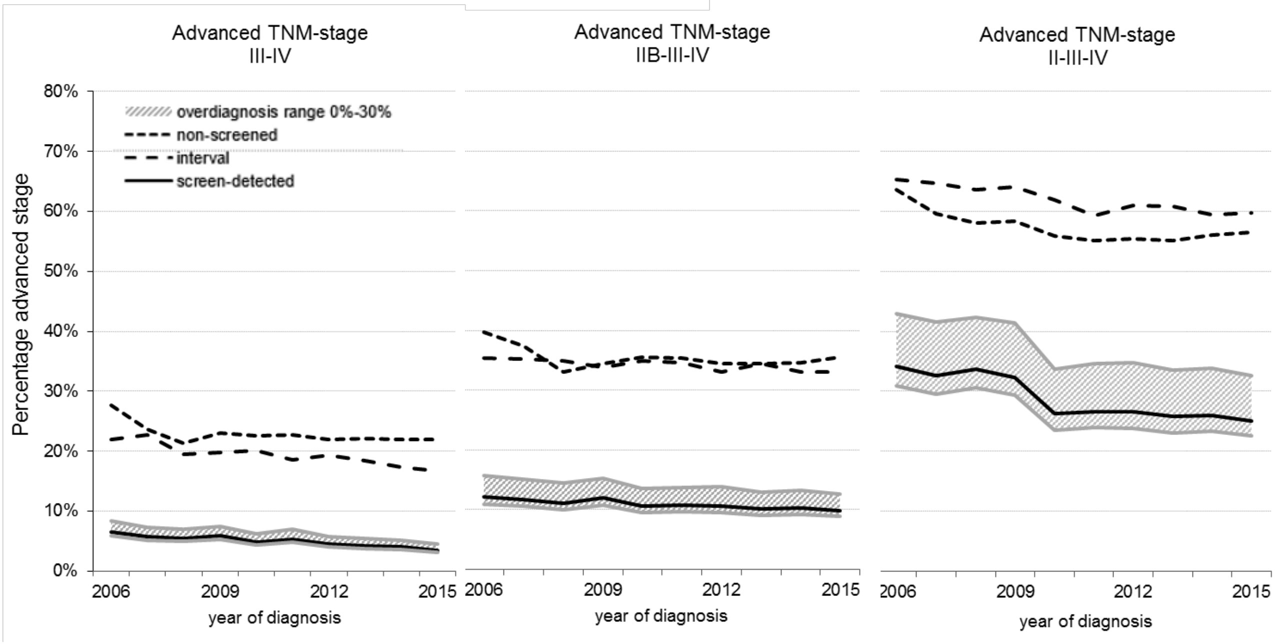

The solid line indicates the screen-detected cancers assuming 10% overdiagnosis. The shaded area then indicates the percentage assuming 0% overdiagnosis (lower limit) to 30% overdiagnosis (upper limit).

Supplementary Figure 2. Odds ratios for advanced breast cancer for two other definitions of advanced TNM-stage, compared to the main definition

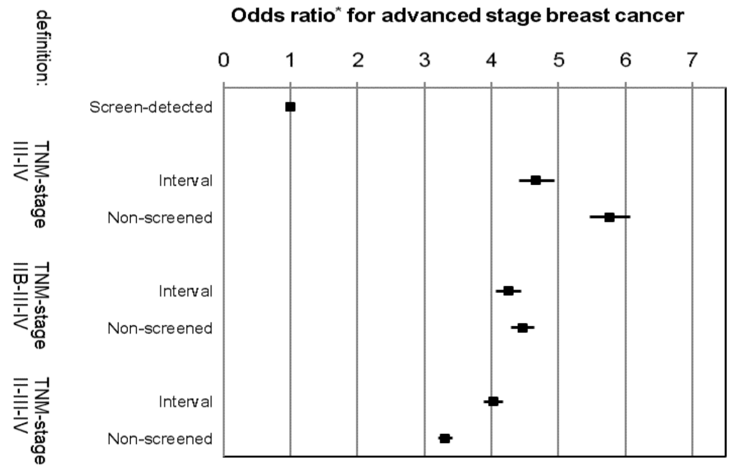

OR	(95% CI)
1	
4.67	(4.41 to 4.97)
5.76	(5.47 to 6.07)
4.25	(4.07 to 4.44)
4.46	(4.29 to 4.65)
4.03	(3.88 to 4.18)
3.42	(3.21 to 3.42)

* Multivariable analyses corrected for age. year of diagnosis. socioeconomic status
Abbreviations: 95%CI: 95% Confidence interval

Supplementary Figure 3. Odds ratios for advanced breast cancer between different cohorts by three definitions of advanced stage, assuming 0%, no overdiagnosis

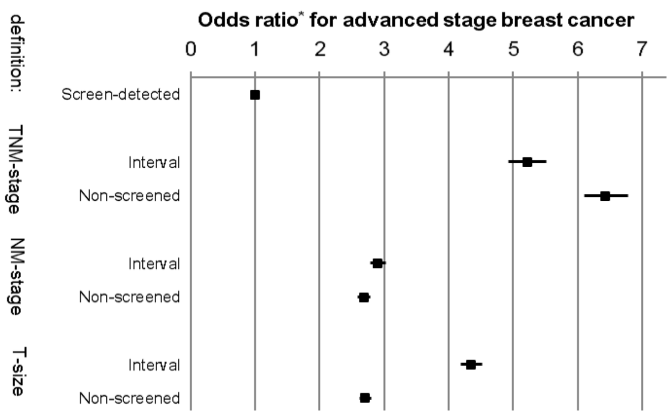

OR	(95% CI)
1	
5.22	(4.93 to 5.52)
6.43	(6.11 to 6.78)
2.91	(2.79 to 3.02)
2.69	(2.59 to 2.78)
4.35	(4.19 to 4.52)
2.71	(2.62 to 2.80)

* Multivariable analyses corrected for age. year of diagnosis. socioeconomic status
Abbreviations: 95%CI: 95% Confidence interval

Supplementary Figure 4. Odds ratios for advanced breast cancer between different cohorts by three definitions of advanced stage, assuming 30% overdiagnosis#

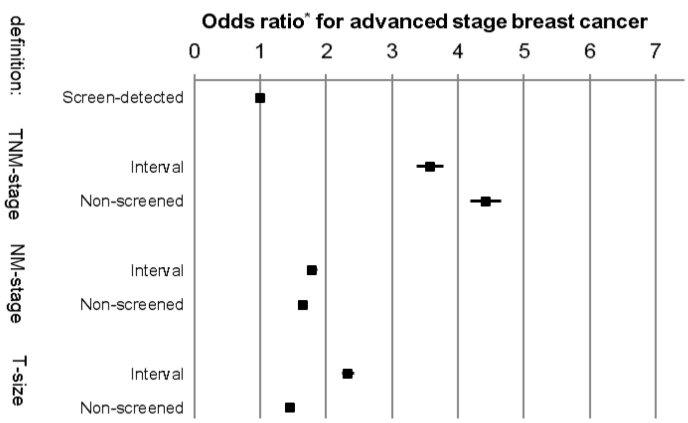

OR	(95% CI)
1	
3.58	(3.38 to 3.79)
4.41	(4.19 to 4.65)
1.79	(1.72 to 1.86)
1.66	(1.60 to 1.72)
2.33	(2.24 to 2.43)
1.45	(1.40 to 1.50)

30% of all screen-detected cancers (n=51,927) were assumed to be overdiagnosed (n=15,578). As overdiagnosis occurs in the early screen-detected cancers by definition. we randomly excluded 15,578 cancers from the early screen-detected cancers as an attempt to correct for overdiagnosis.
* Multivariable analyses corrected for age. year of diagnosis. socioeconomic status
Abbreviations: 95%CI: 95% Confidence interval
